# Relationship between resolution and accuracy of four intraoral scanners in complete-arch impressions

**DOI:** 10.4317/jced.54670

**Published:** 2018-04-01

**Authors:** Priscilla Medina-Sotomayor, Agustín Pascual-Moscardó, Isabel Camps

**Affiliations:** 1 Master, Full Professor, School of Dentistry, Azogues Campus, Universidad Católica de Cuenca, Azogues, Ecuador; 2 Doctor, Full Professor, Department of Dentistry, Universitat de Valencia, Spain; 3 Doctor, Associate Professor, Department of Dentistry, Universitat de Valencia, Spain

## Abstract

**Background:**

The scanner does not measure the dental surface continually. Instead, it generates a point cloud, and these points are then joined to form the scanned object. This approximation will depend on the number of points generated (resolution), which can lead to low accuracy (trueness and precision) when fewer points are obtained. The purpose of this study is to determine the resolution of four intraoral digital imaging systems and to demonstrate the relationship between accuracy and resolution of the intraoral scanner in impressions of a complete dental arch.

**Material and Methods:**

A master cast of the complete maxillary arch was prepared with different dental preparations. Using four digital impression systems, the cast was scanned inside of a black methacrylate box, obtaining a total of 40 digital impressions from each scanner. The resolution was obtained by dividing the number of points of each digital impression by the total surface area of the cast. Accuracy was evaluated using a three-dimensional measurement software, using the “best alignment” method of the casts with a highly faithful reference model obtained from an industrial scanner. Pearson correlation was used for statistical analysis of the data.

**Results:**

Of the intraoral scanners, Omnicam is the system with the best resolution, with 79.82 points per mm2, followed by True Definition with 54.68 points per mm2, Trios with 41.21 points per mm2, and iTero with 34.20 points per mm2. However, the study found no relationship between resolution and accuracy of the study digital impression systems (*P* >0.05), except for Omnicam and its precision.

**Conclusions:**

The resolution of the digital impression systems has no relationship with the accuracy they achieve in the impression of a complete dental arch. The study found that the Omnicam scanner is the system that obtains the best resolution, and that as the resolution increases, its precision increases.

** Key words:**Trueness, precision, accuracy, resolution, intraoral scanner, digital impression.

## Introduction

A digital impression is a three-dimensional (3D) record of the dental structure created using an intraoral scanner. It is the first step of computer-aided design and computer-aided manufacturing (CAD/CAM) systems. These systems refer to the production technique that integrates and applies computer knowledge to both the design and the manufacture of parts. CAD/CAM systems were originally used in engineering, but they are now used in many fields.

The computer translates the information from the scan into a 3D map of the mouth of the patient. The shape of the restoration is then designed, and this design is used by the milling machine to shape the restoration material.

The purpose of a 3D scanner is to generate a point cloud that represents the surface of the object being scanned. The spatial location of these points is defined by their Cartesian coordinates, and these coordinates are used for digital reconstruction of the object.

In every image it takes, the scanner collects information about the distance of each point from the surface of the object within its field of vision. Hundreds of images are made to record the total surface of the object. These are then carried over to a common coordinate reference system, to perform the alignment that involves the fusion of the images to obtain a complete 3D model of the object (1).

Intraoral scanners are optical, use light to perform the measurements, are very fast, and do not distort the scanned surface, except for very shiny and translucent surfaces. These surfaces return light to the scanner by reflection and refraction, which can alter the measurement.

Accuracy in scanning is an essential factor, which influences the survival of the restorations. Within the digital workflow, the dental impression must be able to reproduce the tooth as accurately as possible to aid this survival.

Errors in the scanning protocol include the scanning device that obtains the digital data through a point cloud. To obtain this point cloud, sampling has to be done during the exploration stage, since it cannot represent the entire surface of the cast. The combination of this step and the surface sweep leads to overlapping areas with a high point density or poor areas with a low point density (loss of geometric data information). For example, a loss of data at the cervical margin can introduce a mismatch between the restoration and the prepared tooth (2). This depends on the resolution of the scanner.

**Figure 1:**
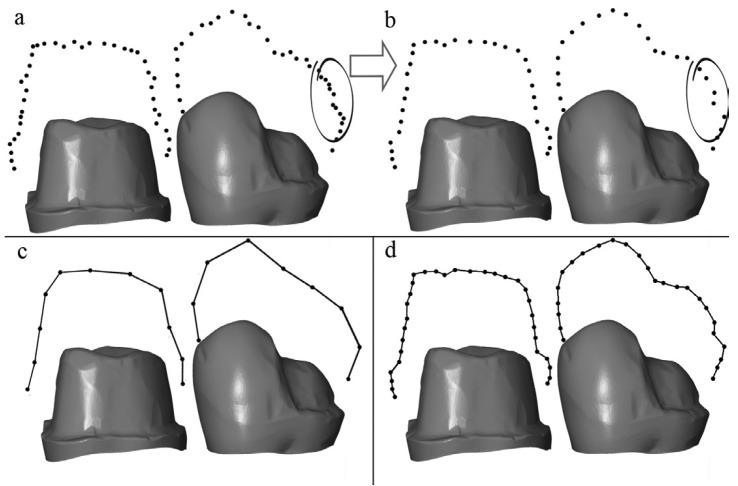
Processing of the point clouds: interpolation and chord error (a) original point cloud (b) filtered point cloud, CHORD ERROR (c) interpolation of a low point density (d) interpolation of a high point density. Experiment performed by Tapia *et al.* (Braunschweig, Germany 2015).

Another error that may occur in the processing of the digital data refers to the processing of points obtained to form the point cloud. Geometric defects can occur in the formatting of the 3D image. This is called chord error (Fig. 1) and may be due to the algorithms used in the process. Scanners with high density (higher resolution) can be converted more easily into a faithful digital image of reality, while those with low density (lower resolution) can introduce errors, due to the lack of digital information between them, which would cause dimensional defects, nonexistent curvatures, or discontinuity in the digital image (2) .

The resolution of a 3D image is the smallest change in a physical magnitude that is being measured and that is able to be detected by the measuring instrument (1). It indicates the quantity of details that can be observed in the image.

**Figure 2:**
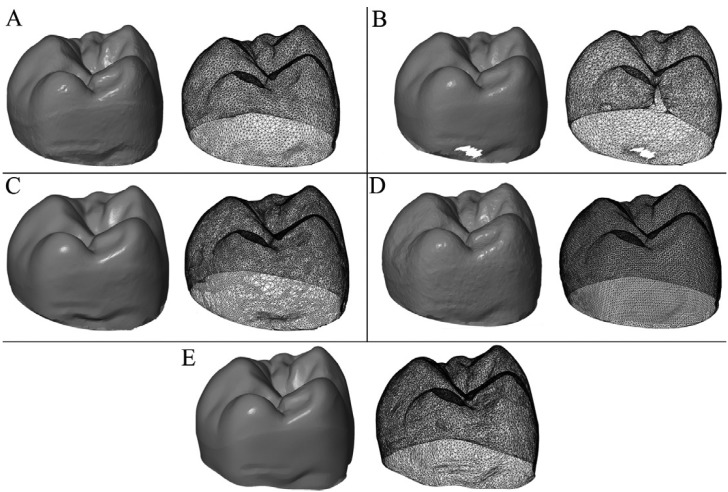
Visual differences in the density of the point cloud (A) Trios (B) iTero (C) Omnicam (D) True definition (E) ATOS Triple Scan II.

In the case of a 3D scanner, this is the number of points that the scanner is able to measure per unit of surface area. If the resolution is higher, the scanner will be able to detect smaller characteristics of an object (Fig. 2).

The scanning device obtains its resolution in relation to the magnitude of volume of the object to be scanned. Therefore, the actual resolution of 3D models can be obtained by dividing the number of points by the surface area. This data will be used to see the relationship between resolution and accuracy of each intraoral scanner.

## Material and Methods

On a cast of a maxillary dental arch, preparations were made for an onlay, veneer, and abutment teeth with supragingival chamfer finish line, leaving the space created between them edentulous. On this cast, four analogs were inserted with Straumann tissue level internal connection, and then a Straumann RN anti-rotational Core3D scanbody was screwed into place (Avinent Implant System, Barcelona, Spain).

This dental cast was then duplicated to prepare the master cast in Exakto-Form epoxy resin (Bredent, Senden, Germany).

Forty scans were performed with each scanner: Trios, iTero, Omnicam, and True Definition ( Table 1).

**Table 1:**
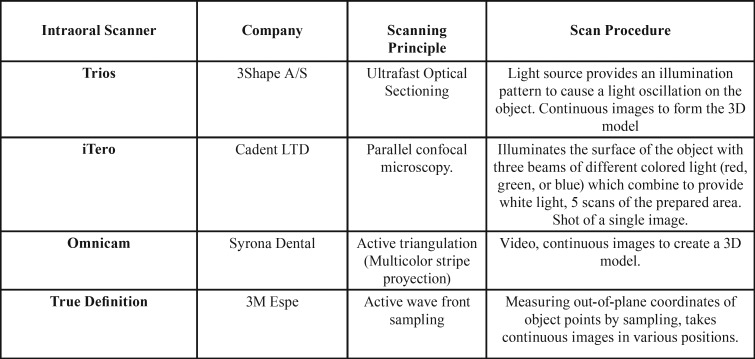
Features of the intraoral impression systems included in the study.

The first three do not require a special preparation of the tooth to be scanned. However, the True Definition scanner requires titanium dioxide, a contrast medium for digital impression, which is used to temporarily add texture to the surface. It is called 3M High-Resolution Scanning Spray Powder and the 3M High-Resolution Sprayer is used to apply this product.

The impressions were taken in an opaque black methacrylate box, to avoid light reflection, and stored in their format.

A “CAD reference model” (CRM) was made (3,4), obtained with ATOS II Triple Scan (GOM Technologies, Metronic, Barcelona, Spain), an industrial structured blue light scanner. This scanner complies with ISO 12836 and certifies a trueness of 3 μ and a precision (repeatability) of 2 μ for scanning of a complete dental arch. (5) With this certainty, a true-to-life version of the master cast was obtained in standard triangle language (STL) format.

The models were standardized in STL files. iTero, Trios, and True Definition scanners export directly in this format. For the Cerec system, Omnicam, Delcam Exchange 2016 R3 software was used.

**Figure 3:**
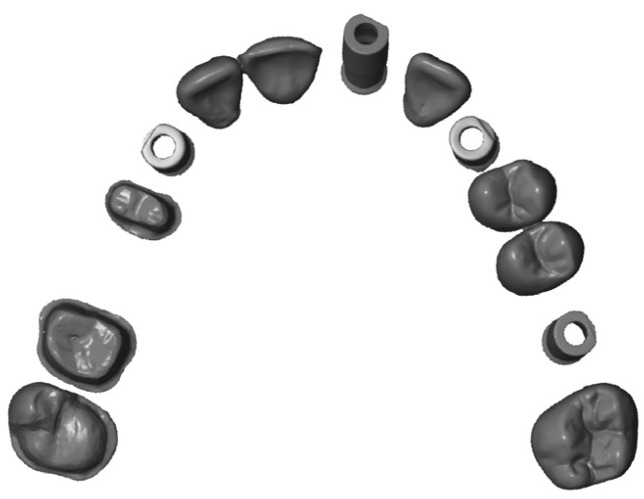
CRM with ATOS II Triple Scan industrial scanner.

Discrepancies were analyzed using Geomagic Control (Geomagic, Morrisville, North Carolina, USA, 2013). (6-10) With its “cut with planes” tool, all of the soft tissue surrounding the teeth was removed, to reduce data points in the file that may affect the mean distance (about half were eliminated) and cutting automation was used so that all models were equal. The digital processing was concluded by smoothing the edges of each study model (Fig. 3).

The study STL files were analyzed, comparing the CRM using the “best fit alignment” method, superimposing the files to calculate the total 3D deviations between the study data sets obtained from the reference scanner and from the different intraoral scanners (trueness and precision) for the complete arch.

This software provides discrepancies in micrometers, both positive (expansion) and negative (contraction).

To obtain the resolution, the number of points of the digital model was divided by the surface area in mm2 of the CRM once cut, data which the software provided; the value obtained was in points/mm (pts/mm).

Trueness was calculated from the average of the mean internal and external discrepancies (expansion and contraction). And precision was obtained from the standard deviation given by the software (repeatability).

Pearson correlation was used to analyze the data, being positive (r=1), negative (r=-1), and no relation (r=0).

## Results

The descriptive data listed in  Table 2 and  Table 3 were analyzed to establish the accuracy, in terms of trueness and precision, as well as the resolution of the intraoral scanners.

**Table 2:**
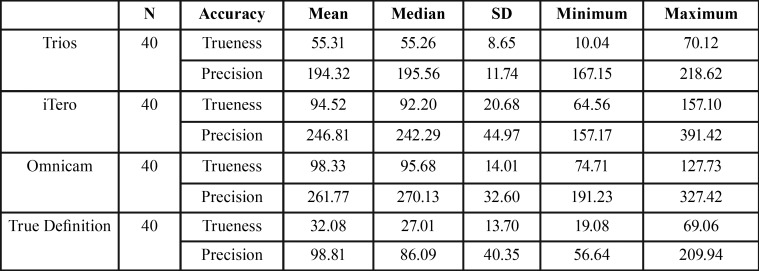
Unprocessed accuracy data (μ), in terms of trueness and precision, used for the statistical analysis of several scanners.

**Table 3:**
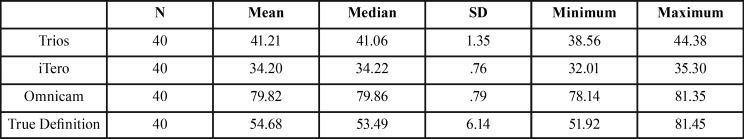
Unprocessed resolution data (pts/mm2) used for the statistical analysis of several scanners.

For the Trios scanner, a resolution of 41.21 pts/mm2 and an accuracy of 55.31 μ +/- 194.53 μ were established; iTero had a resolution of 34.20 pts/mm2 and accuracy of 94.52 μ +/- 246.81 μ and True Definition a resolution of 54.68 pts/mm2 and accuracy of 32.08 μ +/- 98.81 μ ( Table 4).

**Table 4:**
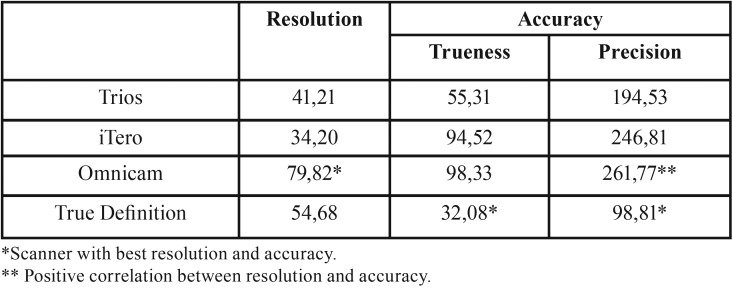
Resolution (pts/mm2) and accuracy (μ), in terms of trueness and precision, of the intraoral scanners on complete dental arch.

None of these scanners demonstrated a relationship between these two variables (r=0).

However, the Omnicam scanner, with a resolution of 79.82 pts/mm2 and accuracy of 98.33 μ +/- 261.77 μ, demonstrated a positive relationship (r=1) between the resolution and its precision values ( Table 4).

## Discussion

Although, due to the different mechanics used for the acquisition of images and the calculation of 3D models, there is no scientific literature that relates the resolution of intraoral scanners to their accuracy, it seems very interesting to analyze the relationship between these variables.

A scanner generates a point cloud, and these points are then joined to form the scanned object. This is caller interpolation. First, the original point cloud is filtered. Then, through interpolation, a likely approximation of what is between these points is produced to form the digital image. All this can cause what is known as chord error, which would be related to the point density (resolution) generated by the intraoral scanner (Fig. 1) (2).

Introducing the variable of resolution attempts analyze the number of points obtained in the digital impression of the complete arch, assuming that the greater the number of points in a certain area, the lower the chord error, due to the greater digital information generated by the scanner.

However, a scanner could produce STL files with a very dense point cloud, and these do not correspond to the actual points of the surface of the physical model. Therefore, the study checked whether this variable reflects better results in the variables of precision and trueness, and whether there is any relationship.

The resolution of each scanner is obtained by dividing the number of total points of each study STL file by the surface area of the cast in 2. The valued obtain is points/mm2. Therefore, it would be conditional to the magnitude of volume of the object to be scanned (1,4,10,11).

Trueness values are calculated from the average of the mean internal and external discrepancies, that is, the mean expansion and contraction that the digital model undergoes compared to the CRM. The precision value corresponds to the standard deviation of the measurements obtained in the study scanners of each group.

In 2002, Rudolph et al (11) introduced a new variable in the measurement of the accuracy of intraoral scanning systems: root mean square (RMS). RMS is the mean deviation between the points that comprise the STL point cloud and is controlled by the resolution of the system. (12). That said, it is assumed that the greater the number of points in a given area, the less chord error the comparison software will introduce in the interpolation of points.

In this study, the resolution of each intraoral digital scanning system was determined in the impression of a complete dental arch ( Table 4), but no correlation was found between a better resolution and the accuracy, in terms of trueness and precision, in a complete dental arch impression on the True Definition, Trios, and iTero scanners, with a resolution of 54.68 (True Definition), 41.21 (Trios), and 34.20 (iTero) pts/mm2.

However, on the Omnicam scanner, which has the best resolution (79.82 pts/mm2) of the study scanners, the study found that the better the resolution (point density) acquired, the better the result in precision of scanning of the object. The low accuracy of this scanner may be due to the adjustment error that occurs in the interpolation of points. Although there is a higher point density, the points are not filtered and ordered appropriately to obtain the 3D image with the trueness of the real model (Fig. 1).

## Conclusions

Within the limitations of this *in vitro* study, it can be concluded that there is no relationship between resolution and accuracy, in terms of trueness and precision, in the True Definition, Trios, and iTero scanner, while the Omnicam scanner does show a positive relationship between resolution and precision in dental impressions of the complete dental arch.
